# Follow-up care by patient's own general practitioner after contact with out-of-hours care. A descriptive study

**DOI:** 10.1186/1471-2296-6-23

**Published:** 2005-06-09

**Authors:** Caro JT van Uden, Paul J Zwietering, Sjoerd O Hobma, Andre JHA Ament, Geertjan Wesseling, Onno CP van Schayck, Harry FJM Crebolder

**Affiliations:** 1 Department of Integrated Care, Research Institute Caphri, University Hospital Maastricht, P.O. Box 5800, 6202 AZ Maastricht, the Netherlands; 2 Department of General Practice, Research Institute Caphri, Maastricht University, P.O. Box 616, 6200 MD Maastricht, the Netherlands; 3 Department of Health Organization Policy and Economics, Research Institute Caphri, Maastricht University, P.O. Box 616, 6200 MD Maastricht, the Netherlands; 4 Department of Respiratory Diseases, University Hospital Maastricht, P.O. Box 5800, 6202 AZ Maastricht, the Netherlands

## Abstract

**Background:**

Little is known about the care process after patients have contacted a GP cooperative for out-of-hours care. The objective of this study was to determine the proportion of patients who seek follow-up care after contact with a GP cooperative for out-of-hours care, and to gain insight into factors that are related to this follow-up care.

**Methods:**

A total of 2805 patients who contacted a GP cooperative for out-of-hours care were sent a questionnaire. They were asked whether they had attended their own GP within a week after their contact with the cooperative, and for what reason. To investigate whether other variables are related to follow-up care, a logistic regression analysis was applied. Variables that entered in this analysis were patient characteristics (age, gender, etc.) and patient opinion on correctness of diagnosis, urgency and severity of the medical complaint.

**Results:**

The response rate was 42%. In total, 48% of the patients received follow-up care from their own GP. Only 20% were referred or advised to attend their own GP. Others attended because their medical condition worsened or because they were concerned about their complaint. Variables that predicted follow-up care were the patient's opinion on the correctness of the diagnosis, patient's health insurance, and severity of the medical problem.

**Conclusion:**

Almost half of all patients in this study who contacted the GP cooperative for out-of-hours care attended their own GP during office hours within a week, for the same medical complaint. The most important factor that predicted follow-up care from the patient's own GP after an out-of-hours contact was the patient's degree of confidence in the diagnosis established at the GP cooperative. Despite the limited generalisability, this study is a first step in providing insight into the dimension of follow-up care after a patient has contacted the GP cooperative for out-of-hours primary care.

## Background

During the last decade, out-of-hours primary care in the Netherlands has been reorganized from practice-based services to large-scale general practitioner (GP) cooperatives [1]. Currently, over 90% of the Dutch population is covered by more than 120 GP cooperatives for out-of-hours primary care. The initiative for this reorganization has come mainly from the medical profession itself. Research has shown that similar reorganisations had beneficial effects in other countries like the UK and Denmark; GPs' satisfaction with out-of-hours services increased and the number of hours the GP has to be on call dropped substantially [2]. Also patients seemed to be fairly satisfied with out-of-hours primary care delivered by GP cooperatives. However, patients seemed to be less satisfied when receiving telephone advice only [3-5].

GP cooperatives, being a new type of organisation in Dutch health care, aim to enhance the efficiency of current care provision. Besides the increased satisfaction of GPs, improved efficiency of the organisation may well be possible. Research into utilisation of out-of-hours services and patient flow can generate relevant insight into functioning of out-of-hours care organisations. Insight into how many patients utilise out-of-hours services, what type of consultation they receive, and which care process follows their contact with the GP cooperative can supply information about the efficiency of the out-of-hours care organisation. Utilisation of out-of-hours services and the type of consultations patients receive have been regularly investigated [2]. However, little is known about the care process after patients have contacted the GP cooperative for out-of-hours care. Only a few studies have included analyses on demand of follow-up care at the patient's own GP's practice, but showed wide variability in numbers and out-of-hours care settings. McKinley et al. [6] found that 54% of all patients who received telephone advice only during out-of-hours (provided by patients' own GP or by commercial deputising services) attended their own GP during office hours with the same problem within two weeks after their out-of-hours contact. For patients who received a home visit this proportion was 45%. Two studies on GPs working at a hospital's emergency department reported that 22 to 26% of the patients went to their own GP within three months after their contact with the GP at the emergency departmen [7,8]. Neither one of these studies, which used patient reports, give insight into how many of these patients were advised to see their own GP for follow-up care, or attended at their own initiative. A study by Shipman et al. [9] showed that GPs working out-of-hours in a practice-based setting referred about 17% of all patients to the patient's own GP the next day. This leaves unknown how many attended at their own initiative.

The purpose of this study is to determine the dimension of follow-up care after contact with a GP cooperative for out-of-hours care, and to gain insight into factors that are related to this follow-up care.

## Methods

The study was conducted in the province of Limburg in the south of the Netherlands. In this province there are seven GP cooperatives operational, which cover a population of about 1.1 million (total population of the Netherlands is approximately 16 million). With respect to out-of-hours primary care, the province is organisationally divided in five regions. Two of these regions each have two cooperatives (NL and ML), one region (OZL) has one GP cooperative with two satellite centres, and in the other two regions (WM and MH) only one GP cooperative is operational. In the year 2002, a total of 307,346 patient contacts with these five organisations were registered. On average, 39% of these contacts were handled with telephone advice only, 51% consisted of a consultation at the GP cooperative, and 10% consisted of home visits. From March to June 2003, a sample of 2805 patients from these five GP cooperative organisations were sent a questionnaire within three weeks after they had been in contact with the GP cooperative. Sampling was performed per out-of-hours care organisation. With respect to patients who received telephone advice only and those who attended the GP cooperative, a computer program selected each fourth patient contact with the out-of-hours primary care organisation backwards from the moment of sampling. Since the number of home visits is limited, all 150 patients, who were visited by a GP from the cooperative prior to the moment of sampling, received a questionnaire. Per region 450 questionnaires were sent out; 150 to patients who received only telephone advice, 150 to patients who visited the GP cooperative, and 150 to patients who received a home visit. Because of parallel research, more questionnaires were sent out in one of the regions (WM): 1005 questionnaires equally distributed among the three types of patient contact with the GP cooperative. Three to four weeks after the questionnaire had been sent, a reminder was sent by mail to patients who had not returned the questionnaire, with the exception of the WM area. This study was part of a larger study on patient satisfaction with out-of-hours primary care [10]. The study was approved by the institutional medical ethics board of the University Hospital Maastricht.

Patients were asked to report whether they had attended their own GP within a week after their contact with the GP cooperative for out-of-hours care for the same medical complaint. They were also asked about their reasons for this attendance.

To investigate whether other variables could predict follow-up care, we also collected information on patient's age and gender, patient's education level (low, medium, high), and health insurances. Health insurance was used as a measure of the patient's socio-economic status: people with an income below a certain amount (some 60% of the population) are compulsorily insured under a public scheme (the Health Insurance Fund). Everyone else has to take out private insurance. Other variables that were collected included: whether the patient thought the diagnosis made by the GP of the cooperative was correct, urgency and severity of the medical complaint (as judged by the patient), patient's concern about his medical condition, whether the patient received the type of consultation (telephone advice, consultation at the GP cooperative, home visit) he or she expected, and the patient's opinion on performance of the GP cooperative on a 10-point scale (1 = very poor and 10 = very good).

To gain insight in how well our study sample represents the study base we collected data on patient gender, age and health insurance. This was done during a four-week period in May and June in 2002 at all GP cooperatives involved in this study.

### Statistics

Descriptive statistics were applied to gain insight into the extent and patients' reasons of follow-up care after contact with the GP cooperative. We performed logistic regression analysis to determine any other factors related to follow-up care. All other variables except for the patient's reason for attendance were entered in the analysis. Variables that did not significantly contribute (*P *> 0.10) to the predictive model were excluded by backward deletion. Only patients, who were not advised or referred to attend their own GP, were included in the logistic regression analysis.

## Results

**Figure 1:**
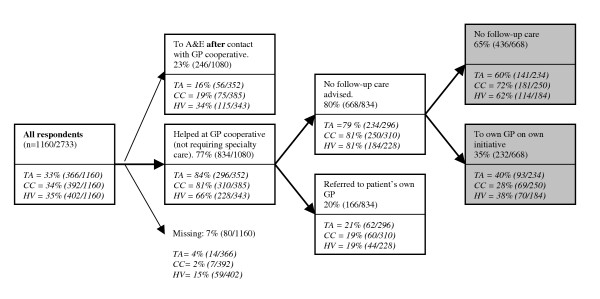
Flow chart of patient follow-up care after contact with the GP cooperative for out-of-hours care. Grey boxes represent data on which the logistic regression has been performed. (TA = telephone advice; CC = consultation at the GP cooperative; HV = home visit; A&E = Accident and Emergency department)

Seventy-two of the 2805 questionnaires were excluded, either because they could not be delivered (patient had moved or had given a false address), the patient had died, or the patient was sent more than one questionnaire (in case of multiple contacts). Eventually the response was 42.4% (1160/2733). Of this group, 834 patients reported to have been helped by the GP or the doctor's assistant of the GP cooperative and did not receive care by a medical specialist at the hospital's emergency department (see figure 1). In total, 47.7% (398/834) of these patients reported to have attended their own GP within a week after their contact with the GP cooperative for the same medical problem. 19.9% (166/834) attended their own GP on advice of the GP or doctor's assistant of the GP cooperative. About one-third of all patients not referred or advised to attend their own GP, still went to see their own GP within a week after their contact with the cooperative at their own initiative.

Patient characteristics of the study sample with respect to the total group are very similar to the responders characteristics, except for age (Table 1). It is known that people who receive home visits are generally older, compared to those patients receiving telephone advice or attending the GP cooperative. Therefore, we also analysed age distribution per type of consultation and found no relevant difference between study base and responders (Table 2).

**Table 1:** Overview of patient characteristics of the study base and responders.

	Study base* (n = 28,535)	Responders (n = 1160)
	n (%)	n (%)

**Gender**		
Male	13,234 (46.4%)	484 (45.5%)
Female	15,288 (53.6%)	579 (54.5%)
Total	28,522 (100%)	1063 (100%)
*missing*	*13 (0.05%)*	*97 (8.4%)*
**Age^#^**		
0–20	7469 (30.0%)	282 (25.1%)
21–40	6548 (26.3%)	203 (18.1%)
40–60	5203 (20.9%)	242 (21.5%)
>60	5677 (22.8%)	396 (35.3%)
Total	24,897 (100%)	1123 (100%)
*missing*	*15 (0.06%)*	*37 (3.2%)*
**Insurance**		
Public	21,875 (78.1%)	865 (76.2%)
Private	6,960 (21.9%)	270 (23.8%)
Total	27,625 (100%)	1135 (100%)
*missing*	*910 (3.2%)*	*25 (2.2%)*

* Based on patient contacts during a four-week period (May – June) in 2002

^# ^Based on 24,897 contacts, excluding one region (WM).

**Table 2:** Age categories of patients contacting the GP cooperative for out-of-hours and age categories of responders in this study.

	Telephone advice		Consultation at the cooperative		Home visits	
	Study base* (n = 5838)	Responders (n = 366)	Study base* (n = 4863)	Responders (n = 392)	Study base* (n = 1305)	Responders (n = 402)

	n (%)	n (%)	n (%)	n (%)	n (%)	n (%)

Age						
0–20 years	1786 (30.6%)	127 (35.5%)	1789 (36.8%)	146 (39.0%)	48 (3.7%)	9 (2.3%)
21–40 years	1587 (27.2%)	96 (26.8%)	1332 (27.4%)	81 (21.7%)	81 (6.1%)	26 (6.6%)
40–60 years	1226 (21.0%)	67 (18.7%)	1099 (22.6%)	82 (21.9%)	231 (17.7%)	93 (23.8%)
>60 years	1237 (21.2%)	68 (19.0%)	642 (13.2%)	65 (17.4%)	944 (72.4%)	263 (67.3%)
**Total**	5836 (100%)	358 (100%)	4862 (100%)	374 (100%)	1304 (100%)	391 (100%)
*missing*	*2 (0.03%)*	*8 (2.0%)*	*1 (0.02%)*	*18 (4.6%)*	*1 (0.08%)*	*11 (2.7%)*

* Based on patient contacts with two cooperatives in the province of Limburg during a four-week period (May – June) in 2002.

### Overall

Of those patients who were not advised or referred to see their own GP within a week for the same problem but attended their own GP anyway, many reported that they had contacted their own GP because of worsening of their medical condition (23%) or because they were worried about their complaint (17%) (see Table 3). In only five percent of the cases, patients reported that wrong advice or treatment was the reason for them to attend their own GP after their contact with the GP cooperative.

**Table 3:** Reasons given by patients for seeking follow-up care with their own general practitioner after their contact with the GP cooperative for out-of-hours care.

	**Telephone advice**	**Consultation at the GP cooperative**	**Home visits**	**Total**
	n (%)	n (%)	n (%)	n (%)

Referred or advised by GP	62 (40.0)	60 (46.5)	44 (38.6)	166 (41.7)
Worsening of complaint	38 (24.5)	30 (23.3)	23 (20.2)	91 (22.9)
Wrong advice or treatment	11 (7.1)	6 (4.7)	3 (2.6)	20 (5.0)
Worried	26 (16.8)	15 (11.6)	26 (22.8)	67 (16.8)
Other reasons	18 (11.6)	18 (14.0)	18 (15.8)	54 (13.6)
Total	155 (100.0)	129 (100.0)	114 (100.0)	398 (100.0)

Besides these reasons, we identified four other variables to predict follow-up care: the patient's opinion on the correctness of the diagnosis, the patient's health insurance, patient satisfaction, and the severity of the medical problem as judged by the patient (Table 5). The model with these four variables was more effective in predicting those who did not attend their own GP within a week: 94.0% of non-attenders and 32.8% of attenders were correctly predicted, with an overall success rate of 73.1%. The overall variance in attendance accounted for was 13% (Cox and Snell test R^2 ^= 0.13).

**Table 5:** Variables related to not-advised follow-up care with the patient's own GP after contact with the GP cooperative as dependent variable (1 = yes, 0 = no). Total group.

**TOTAL GROUP**	B	S.E.	Wald	df	Sig.	Odds ratio Exp(B) (90% C.I.)
INITIAL MODEL*						
Diagnosis	-1.481	0.329	20.200	1	0.000	0.228 (0.132–0.391)
Expectation	-0.440	0.232	3.586	1	0.058	0.644 (0.439–0.944)
Health insurance	0.579	0.292	3.942	1	0.047	1.784 (1.104–2.882)
Gender	-0.123	0.214	0.326	1	0.568	0.885 (0.622–1.259)
Age	0.001	0.005	0.020	1	0.889	1.001 (0.993–1.008)
Patient satisfaction	0.112	0.225	0.248	1	0.619	1.118 (0.773–1.619)
Performance score	-0.024	0.107	0.049	1	0.824	0.977 (0.819–1.164)
Severity	0.486	0.303	2.567	1	0.109	1.625 (0.987–2.676)
Urgency	-0.272	0.291	0.875	1	0.349	0.762 (0.472–1.229)
Worried	0.399	0.332	1.441	1	0.230	1.491 (0.863–2.576)
Education Low			1.142	2	0.565	
Middle	-0.249	0.253	0.964	1	0.326	0.780 (0.514–1.183)
High	-0.177	0.302	0.345	1	0.557	0.838 (0.510–1.376)
Constant	-0.134	1.372	0.010	1	0.922	0.874
						
**FINAL MODEL^#^**						
Diagnosis ^a^	-1.758	0.289	37.078	1	0.000	0.172 (0.107–0.277)
Health insurance ^b^	0.422	0.232	3.320	1	0.068	1.525 (1.042–2.232)
Patient satisfaction ^c^	0.218	0.121	3.236	1	0.072	1.243 (1.019–1.517)
Severity ^d^	0.554	0.222	6.202	1	0.013	1.740 (1.207–2.510)
Constant	-0.403	0.519	0.602	1	0.438	0.669

*31.3% (209/668) missing

^#^13.2% (88/668) missing

^a ^Diagnosis: 1 = right. 0 = wrong.

^b ^Insurance: 1 = public insurance. 0 = private insurance.

^c ^Patient satisfaction (five point scale): 1 representing highly satisfied en 5 very dissatisfied.

^d ^Severity: 1= severe; 0 = not severe.

### Telephone advice

With respect to patients who received telephone advice only, worsening of the complaint and worry about the medical condition were the most frequently reported reasons for patients to attend their own GP without being advised or referred (Table 3). In addition to these reasons, we identified the same three variables to predict follow-up care as for the total sample: correctness of the diagnosis, the severity of the medical problem as judged by the patient, and the patient's health insurance (Table 6). The model with these three variables was also more effective in predicting those who did not attend their own GP within a week: 95.0% of non-attenders and 47.1% of attenders were correctly predicted, with an overall success rate of 74.8%. The variance in attendance accounted for overall was 26% (Cox and Snell test R^2 ^= 0.26).

**Table 6:** Variables related to not-advised follow-up care with the patient's own GP after contact with the GP cooperative as dependent variable (1 = yes. 0 = no). **Telephone advice**

	B	S.E.	Wald	df	Sig.	Odds ratio Exp(B) (90% C.I.)
INITIAL MODEL*						
Diagnosis	-2.471	0.617	16.036	1	0.000	0.085 (0.031–0.233)
Expectation	-0.012	0.407	0.001	1	0.977	0.988 (0.506–1.929)
Health insurance	1.015	0.453	5.030	1	0.025	2.760 (1.311–5.810)
Gender	-0.571	0.388	2.165	1	0.141	0.565 (0.298–1.070)
Age	-0.006	0.009	0.410	1	0.522	0.994 (0.980–1.009)
Patient satisfaction	-0.353	0.374	0.887	1	0.346	0.703 (0.380–1.301)
Performance score	-0.286	0.176	2.637	1	0.104	0.752 (0.563–1.004)
Severity	0.614	0.465	1.743	1	0.187	1.848 (0.860–3.970)
Urgency	-0.189	0.506	0.140	1	0.708	0.827 (0.360–1.902)
Worried	-0.170	0.531	0.103	1	0.748	0.844 (0.352–2.019)
Education Low			1.724	2	0.422	
Middle	-0.567	0.451	1.585	1	0.208	0.567 (0.270–1.190)
High	0.036	0.473	0.006	1	0.939	1.037 (0.476–2.258)
Constant	4.101	2.355	3.031	1	0.082	60.372
						
**FINAL MODEL^#^**						
Diagnosis ^a^	-2.749	0.478	33.081	1	0.000	0.064 (0.029–0.140)
Health Insurance ^b^	0.860	0.388	4.905	1	0.027	2.363 (1.248–4.476)
Severity ^c^	0.578	0.349	2.734	1	0.098	1.782 (1.003–3.166)
Constant	0.863	0.573	2.266	1	0.132	2.369

* 26.5% (62/234) missing

^# ^12.0% (28/234) missing

^a ^Diagnosis: 1 = right. 0 = wrong.

^b ^Insurance: 1 = public insurance. 0 = private insurance.

^c ^Severity: 1 = severe; 0 = not severe.

### Consultation at the GP cooperative

With respect to patients who went to the GP cooperative for consultation, again, worsening of the complaint and worry about the patient's medical condition were reported as most important reasons to attend the patient's own GP, without being advised or referred. Besides these reasons, we identified two variables to predict follow-up care: correctness of the diagnosis, and the patient's concern about his or her medical problem (Table 7). The model with these two variables was more effective in predicting those who did not attend their own GP within a week: 96.4% of non-attenders and 25.0% of attenders were correctly predicted, with an overall success rate of 76.8%. However, the overall variance in attendance accounted for was small (Cox and Snell test R^2 ^= 0.11).

**Table 7:** Variables related to not-advised follow-up care with the patient's own GP after contact with the GP cooperative as dependent variable (1 = yes. 0 = no). Consultation at the GP cooperative.

CONSULTATION AT GP COOPERATIVE	B	S.E.	Wald	df	Sig.	Odds ratio Exp(B) (90% C.I.)
INITIAL MODEL*						
Diagnosis	-1.934	0.662	8.525	1	0.004	0.145 (0.049–0.430)
Expectation	0.502	0.660	0.577	1	0.447	1.652 (0.557–4.894)
Health insurance	-0.038	0.555	0.005	1	0.945	0.962 (0.386–2.397)
Gender	-0.079	0.418	0.036	1	0.850	0.924 (0.464–1.839)
Age	0.003	0.010	0.106	1	0.745	1.003 (0.987–1.020)
Patient satisfaction	0.211	0.486	0.188	1	0.664	1.235 (0.555–2.745)
Performance score	0.035	0.239	0.021	1	0.884	1.035 (0.699–1.534)
Severity	0.549	0.549	0.999	1	0.318	1.732 (0.701–4.274)
Urgency	-0.531	0.513	1.072	1	0.300	0.588 (0.253–1.367)
Worried	1.014	0.689	2.165	1	0.141	2.757 (0.887–8.566)
Education Low			0.341	2	0.843	
Middle	0.244	0.510	0.229	1	0.632	1.277 (0.551–2.957)
High	-0.130	0.565	0.053	1	0.818	0.878 (0.347–2.223)
Constant	-1.564	3.021	0.268	1	0.605	0.209
						
**FINAL MODEL^#^**						
Diagnosis ^a^	-2.075	0.484	18.359	1	0.000	0.126 (0.057–0.279)
Worried ^b^	1.073	0.520	4.266	1	0.039	2.925 (1.244–6.876)
Constant	-0.083	0.651	0.016	1	0.899	0.920

*33.6% (84/250) missing

^# ^6.8% (17/250) missing

^a ^Diagnosis: 1 = right. 0 = wrong.

^b ^Worried: 1 = worried about own medical complaint. 0 = not worried.

### Home visits

Similar to patients who received telephone advice only or visited the GP cooperative for consultation, patients who received a home visit reported that worry about their medical problem and worsening of the complaint were the main reasons to contact their own GP within a week after their contact with the GP cooperative. Only 3 patients (2.7%) said to have attended their own GP within a week because they believed to have received incorrect advice or treatment by the home visiting GP of the GP cooperative.

We also investigated other variables and their relationship with follow-up care, but none of the variables entered in the logistic regression analysis was able to predict whether the patient did or did not attend his or her own GP within one week after they had been visited by the GP of the cooperative at home (Table 8).

**Table 8:** Variables related to not-advised follow-up care with the patient's own GP after contact with the GP cooperative as dependent variable (1 = yes. 0 = no). Home visits.

**HOME VISITS**	B	S.E.	Wald	df	Sig.	Odds ratio Exp(B) (90% C.I.)
INITIAL MODEL*						
Diagnosis	0.468	0.752	0.388	1	0.533	1.597 (0.464–5.503)
Expectation	-1.134	0.631	3.228	1	0.072	0.322 (0.114–0.909)
Health insurance	0.507	0.749	0.458	1	0.499	1.661 (0.484–5.695)
Gender	0.550	0.440	1.567	1	0.211	1.734 (0.841–3.572)
Age	-0.001	0.012	0.003	1	0.959	0.999 (0.980–1.019)
Patient satisfaction	0.990	0.508	3.801	1	0.051	2.691 (1.167–6.202)
Performance score	0.491	0.252	3.801	1	0.051	1.634 (1.080–2.473)
Severity	0.881	1.228	0.515	1	0.473	2.414 (0.320–18.198)
Urgency	1.007	0.944	1.139	1	0.286	2.738 (0.580–12.932)
Worried	0.320	0.813	0.155	1	0.694	1.377 (0.362–5.240)
Education Low			3.522	2	0.172	
Middle	-0.161	0.455	0.125	1	0.724	0.851 (0.402–1.801)
High	-1.868	0.999	3.496	1	0.062	0.154 (0.030–0.799)
Constant	-9.070	3.334	7.402	1	0.007	0.000
						
**FINAL MODEL**						
-	-	-	-	-	-	-

* = 34.2% (63/184) missing

## Discussion

This study showed that almost half of all respondents received follow-up care at their own GP's practice, within a week, for the same medical complaint for which they had contacted the GP cooperative. Although a substantial number of these patients (40%) were referred or advised by the cooperative's GPs or doctor's assistants to do so, about 60% of these patients attended their own GP at their own initiative.

According to the Statistics Netherlands Database over the last four years, about 27% of all patients require follow-up care after they have seen their GP for a medical complaint [11]. The fact that in this study substantially more patients received follow-up care can be explained by several factors. First, the medical complaints presented during out-of-hours may be more severe compared to during office hours, and therefore require follow-up care more often. Second, the GP cooperative focuses mainly on medical complaints that cannot wait until the next day; all other non-urgent disorders are often referred to the patient's own GP the next day. Third, patients may not feel fully confident or are not fully satisfied with the way their complaint has been taken care of and want to check this with their own GP. Since we have not compared the situation during office hours with outside office hours, it remains unclear which of these factors contributes the most to the difference in numbers of follow-up care.

In addition to the fact that patients have reported to attend their own GP at their own initiative mainly because their medical condition worsened or that they were worried, we found that generally three other variables were related to follow-up care. The most important variable was whether the patient believed that the correct diagnosis had been made by the cooperative's GP or doctor's assistant. However, this variable might have been biased when the patient's own GP made another diagnosis than either the cooperative's GP or doctor's assistant, since patients may have more confidence in their own GP than in an unknown GP or doctor's assistant. We also found that health insurance was a predictor of follow-up care without referral. This may be explained by the fact that privately insured patients may not be fully reimbursed for these consultations. This implies some kind of financial incentive. In addition, research has shown that patients with lower socio-economic status more frequently attend their GP [12], which is in line with our findings.

For those who received a home visit, no model could be established that predicts follow-up care at the patient's own GP cooperative. Regarding patients who received telephone advice only, or attended the GP cooperative, we found that the correctness of the diagnosis as judged by the patient was a strong predictor for follow-up care. However, many patients who received home visits will already have a known diagnosis, which may give this variable limited predictive value in this patient category. Furthermore, for patients who received a home visit the patient's own GP may often take the initiative to visit the patient, possibly because of the severity of the complaint or co-morbidity, instead of the patient taking the initiative to contact the GP. Patients receiving home visits often suffer from more severe conditions and are significantly older than those helped by telephone advice and those who visited the GP cooperative. Also these two factors may give some explanation for not finding a model to predict re-attendance, because it is known that elderly people more frequently contact the GP and that the GP routinely visits the patient to check on his or her condition.

An important limitation of the study is that the response rate to the questionnaire was only 42.4%. Therefore, care should be taken with generalising these results to all patient contacts with GP cooperatives. It could be that the number of patients seeking follow-up care in this study has been overestimated or underestimated. However, the proportion of patients who went to their own GP for the same complaint was similar to that reported in the literature [6]. In addition, the number of patients who attended their own GP for the same medical complaint on advice of the cooperative's GP or doctor's assistant reported in this study (19%), was fairly similar to that reported by Shipman *et al*. [9] (17%).

This study did not provide insight into appropriateness of follow-up care, but is merely a first step in revealing the extent of follow-up care after contact with a GP cooperative. Therefore, it remains unclear whether the proportion of patients who seek follow-up care by their own GP is appropriate or represents inefficient care. The latter meaning that too many patients require follow-up care because they believed that care at the GP cooperative was insufficient. Future research is warranted to confirm our study findings and to investigate the appropriateness of follow-up care.

## Conclusion

This study is a first step in providing insight into the dimension of follow-up care after a patient has contacted the GP cooperative for out-of-hours primary care. We showed that about half of all respondents who contacted the GP cooperative attended their own GP for the same medical problem within a week. Only a minority of the patients was referred or advised to do so. Most cited reasons were worsening of and worry about the complaint. With respect to those that attended their own GP for the same problem on their own initiative, the perception that the correct diagnosis had been made at the GP cooperative was a strong predictor of non-attendance.

## Competing interests

The author(s) declare that they have no competing interests.

## Authors' contributions

CU participated in the design of the study, performed the statistical analysis, and drafted the manuscript. PZ, SH, AA, GW, CS, and HC participated in the design of the study, supervised the project, and provided critical edits to this manuscript.

**Table 4:** Characteristics of patients not advised for follow-up care with their own general practitioner.

	**Telephone advice (n = 234)**	**Consultation at the GP cooperative (n = 250)**	**Home visits (n = 184)**	**Total (n = 668)**
	
	n (%)	n (%)	n (%)	n (%)
Gender				
Male	92 (40.9)	98 (48.5)	87 (49.2)	277 (45.9)
Female	133 (59.1)	104 (51.5)	90 (50.8)	327 (54.1)
*Missing*	*3.8%*	*19.2%*	*2.7%*	*9.6%*
Age (years)	30.7 (SD 24.2)	29.0 (SD 23.8)	63.5 (SD 19.1)	39.2 (SD 27.3)
*Missing*	*2.1%*	*6.4%*	*2.7%*	*3.9%*
Education level				
Low	55 (25.2)	49 (21.0)	79 (48.8)	183 (29.9)
Average	111 (50.9)	125 (53.6)	60 (37.0)	296 (48.3)
High	52 (23.9)	59 (25.3)	23 (14.2)	134 (21.9)
*Missing*	*6.8%*	*6.8%*	*12.0%*	*8.2%*
Health insurance				
Public	165 (71.1)	182 (74.3)	151 (83.4)	498 (75.7)
Private	67 (28.9)	63 (25.7)	30 (16.6)	160 (24.3)
*Missing*	*0.9%*	*2.0%*	*1.6%*	*1.5%*
				
Received type of consultation as expected				
Yes	84 (38.0)	206 (84.8)	151 (85.8)	441 (68.9)
No	137 (62.0)	37 (15.2)	25 (14.2)	199 (31.1)
*Missing*	*5.6%*	*2.8%*	*4.3%*	*4.2%*
Patient's perceived severity of complaint				
Not severe	87 (37.8)	85 (34.7)	9 (5.1)	181 (27.8)
Severe	143 (62.2)	160 (65.3)	168 (94.9)	471 (72.2)
*Missing*	*1.7%*	*2.0%*	*3.8%*	*2.4%*
Patient's perceived urgency of complaint				
Not urgent	80 (35.4)	62 (25.4)	13 (7.4)	155 (24.0)
Urgent	146 (64.6)	182 (74.6)	162 (92.6)	490 (76.0)
*Missing*	*3.4%*	*2.4%*	*4.9%*	*3.4%*
Patient's worry about complaint				
Not worried	36 (15.5)	43 (17.7)	16 (9.1)	95 (14.6)
Worried	196 (84.5)	200 (82.3)	159 (90.9)	555 (85.4)
*Missing*	*0.9%*	*2.8%*	*4.9%*	*2.7%*
Patient's perceived correctness of diagnosis				
Not correct	47 (22.4)	25 (10.5)	18 (12.0)	90 (15.0)
Correct	163 (77.6)	214 (89.5)	132 (88.0)	509 (85.0)
*Missing*	*10.3%*	*4.4%*	*18.5%*	*10.3%*
Patient satisfaction	2.43 (SD 0.96)	2.03 (SD 0.70)	1.99 (SD 0.87)	2.16 (SD 0.87)
*Missing*	*0.9%*	*0%*	*1.1%*	*0.6%*
Performance score	6.7 (SD 2.0)	7.7 (SD 1.4)	7.6 (SD 1.8)	7.3 (SD 1.8)
**Missing**	*5.6%*	*2.8%*	*4.3%*	*4.2%*

## References

[B1] Van Uden CJT, Winkens RAG, Wesseling GJ, Crebolder HFJM, Van Schayck CP (2003). Use of out of hours services: a comparison between two organisations. Emerg Med J.

[B2] Leibowitz R, Day S, Dunt D (2003). A systematic review of the effect of different models of after-hours primary medical care services on clinical outcome, medical workload, and patient and GP satisfaction. Fam Pract.

[B3] Pickin DM, O'Cathain A, Fall M, Morgan AB, Howe A, Nicholl JP (2004). The impact of a general practice co-operative on accident and emergency services, patient satisfaction and GP satisfaction. Fam Pract.

[B4] Salisbury C (1997). Postal survey of patients' satisfaction with a general practice out of hours cooperative. BMJ.

[B5] Shipman C, Payne F, Hooper R, Dale J (2000). Patient satisfaction with out-of-hours services; how do GP co-operatives compare with deputizing and practice-based arrangements?. J Public Health Med.

[B6] McKinley RK, Cragg DK, Hastings AM, French DP, Manku-Scott TK, Campbell SM, Van F, Roland MO, Roberts C (1997). Comparison of out of hours care provided by patients' own general practitioners and commercial deputising services: a randomised controlled trial. II: The outcome of care. BMJ.

[B7] Dale J, Lang H, Roberts JA, Green J, Glucksman E (1996). Cost effectiveness of treating primary care patients in accident and emergency: a comparison between general practitioners, senior house officers, and registrars. BMJ.

[B8] Murphy AW, Bury G, Plunkett PK, Gibney D, Smith M, Mullan E, Johnson Z (1996). Randomised controlled trial of general practitioner versus usual medical care in an urban accident and emergency department: process, outcome, and comparative cost. BMJ.

[B9] Shipman C, Dale J (1999). Responding to out-of-hours demand: the extent and nature of urgent need. Fam Pract.

[B10] Van Uden CJT, Ament AJHA, Hobma SO, Zwietering PJ, Crebolder HFJM (2005). Patient satisfaction with out-of-hours primary care in the Netherlands. BMC Health Serv Res.

[B11] Statline. http://www.cbs.nl/en/.

[B12] Kolk AM, Hanewald GJ, Schagen S, Gijsbers van Wijk CM (2002). Predicting medically unexplained physical symptoms and health care utilization. A symptom-perception approach. J Psychosom Res.

